# "Hospital utilization by Mexican migrants returning to Mexico due to health needs"

**DOI:** 10.1186/1471-2458-11-241

**Published:** 2011-04-18

**Authors:** Miguel A González-Block, Luz A de la Sierra-de la Vega

**Affiliations:** 1 Centre for Health Systems Research, National Institute of Public Health, Av. Universidad 655 Col. Santa María Ahuacatitlán. Cuernavaca, Morelos, 62100, México

## Abstract

**Background:**

A total of 12.7 million Mexicans reside as migrants in the United States, of whom only 45% have health insurance in this country while access to health insurance by migrants in Mexico is fraught with difficulties. Health insurance has been shown to impact the use of health care in both countries. This paper quantifies hospitalizations by migrants who return from the US seeking medical care in public and private hospitals in the US-Mexico border area and in communities of origin. The proportion of bed utilization and the proportion of hospitalizations in Mexico out of the total expected by migrants in the US were estimated.

**Methods:**

The universe included 48 Ministry of Health and 47 private hospitals serving municipalities of high or very high migration in Mexico, where 17% of remittance-receiving households are located, as well as 15 public and 159 private hospitals in 10 Mexican cities along the border with the US. Hospitals were sampled through various methods to include 27% of beds. Patients and staff were interviewed and data triangulated to quantify migrants that returned to Mexico seeking medical care. Official hospital discharge statistics and secondary data from migration databases and published statistics were analyzed to identify bed occupancy, general migrant hospitalization rates and the size of the migrant population that maintains close relationships with households in communities of origin.

**Results:**

Up to 1609 migrants were admitted to public hospitals (76.6%) and 492 to private hospitals (23.4%) serving municipalities of high and very high migration intensity in 2008. Up to 0.90% of public hospital capacity was used. In the border area up to 908 and 2416 migrants were admitted to public (27.3%) and private (72.7%) hospitals, respectively. Up to 1.18% of public hospital capacity was used. Between 2.4% and 20.4% of the expected hospitalization needs of migrants with dependent households are satisfied through these services. The most common diagnostic categories mentioned across hospitals were traumatisms, complications of diabetes and elective surgery, in that order. Private hospitals mention elective surgeries as the main diagnostic category followed by complications of diabetes.

**Conclusions:**

Hospitals in communities of origin in Mexico are devoting few resources to respond to hospitalization needs of migrants in the US. Currently no hospital programs exist to stimulate migrant demand or to cater to their specific needs. Registering migratory history in clinical and administrative records can be readily implemented. Developing bi-national referral networks and insuring migrants in the US within current Mexican federal programs could greatly increase migrant access to hospitals.

## Background

Close to 12% of all Mexican citizens or 12.7 million currently reside in the United States enjoying or enduring diverse migratory statuses. The majority are long-stay migrants, with 68% having over 10 years of residence [1]. The migrants' flow is continuous, with up to 700,000 crossing the border northward and 250,000 returning every year [2]. While 21% have dual citizenship, up to 60% lack migratory papers. In general, the population of Hispanic origin in the U.S. enjoys better health than the average, with the most important exception being diabetes, which is higher among Mexican migrants [3]. Furthermore, evidence indicates that migrants' health status tends to worsen as they assimilate to local life style [4]. The case has also been made that migrants are exposed to a wider range of hazards in the US due to occupational factors [5].

Undocumented Mexicans migrants report less use of health care services in the United States as well as poorer quality of care compared with their US-born counterparts [6]. However, migrants of Mexican origin in the United States are more likely to be hospitalized than non-migrants in Mexico, regardless of health insurance. Indeed, while the general hospital discharge rate in the four states of high migration intensity of Guanajuato, Jalisco, Michoacan and Zacatecas was 3.6% in 2005 [7], in the US the rate among Hispanics with low English proficiency -the vast majority migrants of Mexican origin- was of 6.8% for 2008 [8].

Several studies document the utilization of medical and dental services by the US population of Mexican origin in Mexico, demonstrating the demand for quality, lower cost interventions and medicines [9]. According to Wallace et al [10], 6.2% of long-stay (≥15 years) and 5.2% of short-stay Mexican migrants resident in California demanded medical care in Mexico in 2001. The strongest predictor of medical service use in Mexico was by far lack of health insurance (Odds ratio 4.61). Poverty was not associated to the use of medical care. Studies of willingness to pay for cross-border health insurance show that 57% of migrants would demand a hypothetical product costing between USD 75 and USD 125 per month. Community Health Centers in the US have shown interest in delivering insured primary health care products for migrants, in combination with insured hospital care in Mexico [11].

In Mexico, the Ministry of Health (MoH) has increased health financing for the self-employed and the uninsured laboring in the informal sector of the economy through the "Seguro Popular" policy. Seguro Popular aims to reduce out-of-pocket expenditures by providing a free-at-point-of service, defined package of health benefits to individuals who voluntarily affiliate through a means-tested prepayment. While the government is on track to reach universal coverage through Seguro Popular for 2011, it has acknowledged that "a small number" may not affiliate due to lack of interest [12]; migrants are often included in this group. Indeed, Mexican migrants in the US are not considered within the potential population to be insured by Seguro Popular. Non-the-less, limited efforts have been made to pre-affiliate Mexican nationals visiting consulates in the US as a part of health promotion efforts. Other long-standing efforts to affiliate migrants to mainstream social security health insurance in Mexico have met with very poor results [13].

The strong ties of migrants to their original households in communities of origin could favor bi-national insurance schemes: 85% of migrants send remittances to 1.3 million Mexican homes, while 16% of married men leave their wives behind. Studies suggest that between 10% and 30% of remittances are spent on health care in Mexico [13]. Demand for care in Mexico, close ties to communities of origin and the volume of health funding spent by migrants represent opportunities to adapt Seguro Popular or other insurance schemes to the needs of migrants in the US.

This paper aims to provide evidence on the need for bi-national health insurance to cover hospital care by migrants in Mexico. Research measured the order of magnitude of public and private hospitalizations in Mexico of migrants who returned specifically from the US for this purpose. Two specific settings in Mexico were analyzed: the US-Mexico border region and migrants' communities of origin in municipalities of high and very high migration intensity (interior region). Based on data for the latter, the share of discharges in communities of origin in Mexico out of the total expected by migrants in the US was estimated.

## Methods

### Design and study population

This is a cross-sectional study based on patient and staff surveys to estimate frequency of hospitalizations of repatriated migrants occurring in Mexican private and Ministry of Health hospitals and the proportion with respect to all hospitalizations in same hospitals and with respect to all expected migrant hospitalizations in the last year (Table 1). Hospitalizations were measured for two regions in Mexico: the interior region including all municipalities of high and very high migration intensity and the border region including the ten US-Mexico border cities.

**Table 1:** Sampling Framework and Sample for Hospitals

REGION	States	Municipalities	Hospitals				Beds			
			
			MoH		Private	Total	MoH		Private	Total
								
			General	Referral			General	Referral		
**COMMUNITIES OF ORIGIN**										
Very high migration states*										
Total in universe	5	18	18	5	40	**63**	551	1615	627	**2793**
Sample	4	19	15	4	14	**33**	360	1202	268	**1830**
Other states**										
Total in universe	11	15	15	10	7	**32**	642	1285	71	**1998**
Sample	7	10	10	0	0	**10**	556	0	0	**556**
BORDER***										
Total in universe	5	10	10	5	159	**174**	819	701	2205	**3725**
Sample	3	6	6	0	6	**12**	610	0	211	**821**

**TOTAL**										
Total in universe	**21**	**43**	**43**	**20**	**206**	**269**	**2012**	**3601**	**2903**	**8516**
Sample	**14**	**35**	**31**	**4**	**20**	**55**	**1526**	**1202**	**479**	**3207**

* Nayarit excluded given it had no hospitals located in municipalities of high and very high migration.

** Includes only hospitals in municipalities with high and very high migration intensity

***Nuevo León excluded given it had no hospitals located in border municipalities

Selection of municipalities for the interior region was based on the Mexican Population Council's Index of Migration Intensity, which groups household according to a composite census measure of a) reception of remittances; b) having at least one member living in the US in the past five years; c) with migrants planning to return to the US, and d) with returned migrants in the past five years [14]. Out of 2454 municipalities in Mexico, 27 are classified as of high migration intensity and 6 as of very high migration intensity. These 33 municipalities accounted in 2000 for remittances from 2.4 million migrants (32% of the total) and for 17% of the households receiving them.

A census was done of all general MoH hospitals serving the interior region, including 33 general hospitals (916 beds) in 16 states and 33 municipalities [7]. Referral hospitals in the interior region were observed only for the capital cities of states classified as a whole as of very high migration intensity: Guanajuato, Jalisco, Michoacan and Zacatecas. Private hospitals were sampled from a register of 206 hospitals and 2903 beds [15]. The largest private hospital was selected within each high and very high migration intensity municipality of the same four states. In the border region, all public hospitals -one per city- were selected as well as the largest private hospital.

Some hospitals were excluded at the outset from the study in the interior region states of Durango, Oaxaca and State of Mexico and in the border states of Chihuahua and Coahuila. These states could not be visited due to safety restrictions stemming from widespread violence or political turmoil (3 states), refusal by state authorities to access hospitals (1 state), cost restrictions (1 state) or lack of general hospitals (1 state).

### Outcome measures and definitions

The outcome measure sought was hospitalizations in Mexico by migrants that were repatriated due to illness: defined as migrants with recent illness or medical need in the US that were forced to return to Mexico to take care of their illness or medical need. The share of hospitalizations by repatriated migrants out of the total in the same hospitals was estimated as a measure of the burden of care. The share of repatriated hospitalizations out of the total expected migrant hospitalizations in the US and Mexico was also estimated. To make this measurement possible, the study estimated the universe of migrants susceptible to repatriate for health reasons to the interior region. Two separate assumptions were made for this estimate: that migrants will repatriate only if they have households that will welcome them, and that these households are more likely to welcome them if they receive remittances.

To measure frequency of repatriate hospitalizations a three-stage questionnaire was applied by trained interviewers to all patients in selected hospitals during a one-day visit. The first stage identified patients with a visit to or residence in the US in the past year. The second stage identified patients that presented their current main illness in the US. The third stage requested for these patients demographic data, health care history in the US and in Mexico for the current main illness, migratory history, reasons for repatriation and means of referral. Medical records were inspected to ascertain diagnoses. Informed consent was applied in all cases and the response rate was 100%.

Patient data was triangulated with a semi-structured interview to hospital staff in charge of social work and to the hospital director and to other staff, as available. The hospital social worker and director were interviewed together, including in some cases the deputy director. In half of all interviews it was possible to include one of the following department heads: administration, emergency services, surgery or training. All participants provided informed consent. Care was taken to clearly explain and confirm understanding of the patient inclusion criteria and to elicit information from memory in as reliable a manner as possible.

The interviews requested information on the number of repatriated migrants cared for in the hospital in the previous year (2008), their most prevalent diagnoses and referral patterns. Staff was first asked to focus on the migrant population they cared for as a whole and to conceptually separate returned migrants who presented their illness or health need in Mexico from those that returned due to illness or health needs presented in the US. Staff was asked to consider both elective surgery patients and those admitted for other conditions. Staff were then asked to elicit both the absolute number of migrants that were repatriated due to illness or need and the proportion that were hospitalized out of the total. No distinction was made between hospitalized persons and admissions, assuming that the number of migrants with more than one admission in the past year was insignificant. They were also asked to report on seasonal and yearly fluctuations. All social work staff responded they were able to identify migrants with these conditions with relative ease. All interviews here held in the months immediately after the high season to enable staff to identify the yearly migrant admissions.

### Statistical analysis

Direct patient data was annualized for the sample hospitals through estimating average bed-days for interior and border region hospitals through the following formula:

Where:

M_h _= Number of migrants discharged annually in sample hospitals within the region.

m = interned migrants in sample hospitals observed during one day.

d = average admission days in the hospitals in the region for 2008.

Sample hospital data was expanded for the universe of comparable hospitals in the region. Only similarly sized private hospitals were included in the universe, and referral hospital data was used only to expand to other referral hospitals. The following formula was used:

Where:

M_H _= total repatriated migrants admitted in all hospitals in the region

HDH = admissions for all hospitals of the region for 2008

HDh = admissions for all hospital in the sample in 2008.

Bed-stay data was available from official registers only for public hospitals [7]. Private hospital bed-days were estimated based only on staff reports for total yearly admissions. No direct surveys were applied to patients in private hospitals due to lack of data to estimate annual case-load.

The frequency of interior region household members that have travelled recently to the US or Canada was estimated as a proxy of the number of migrants likely to return for health reasons. The Mexican Migration Project (MMP) database [16] was processed for this purpose, using data for the 7 states of high or very high migration from 2000 to 2009 on the percentage of households and number of household members that report travel to the US. It was found that 54.8% of households are dependent on at least one recent migrant, and that each household is dependent on 1.4 recent migrants on average. The migrant household dependency factor was therefore estimated as 0.767 (54.8% × 1.4).

The hospitalization rate for the migrant population in the US over 18 years of age was taken to be 6.8%, taken from Brach & Chevarley [8] and based on Hispanic respondents with limited English proficiency to the Medical Expenditure Panel Survey (MEPS) of 2004. The uninsured in this population is 59.6%, almost identical to the figure for Mexican migrants and whose socioeconomic indicators correspond in general very well to those of Mexican migrants as known from other studies [1].

Stata v. 10 was used for statistical analysis and Atlas ti v. 5.0 for qualitative analysis.

## Results

Hospitals visited in the study accounted for 75.8% of public beds in the universe and 16.5% of private beds (Table 1). A total of 1,743 patients -all those registered in hospital ledgers- were interviewed during hospital visits: of them, 25 had lived in the US in the last year. Out of all patients contacted, 12 met the inclusion criteria of the current illness as the main reason of returning to the country: 4 in the border region and in the interior 7 in general hospitals and 1 in a referral hospital (Table 2).

**Table 2:** Annualized Sample and Expanded Data for Mexican Hospital Discharges of Migrants With Recent Migratory History or Repatriated Sick from the US, by Region and Type of Care, 2008

REGION		MoH		Private		Total	
		
		Sample	Expanded	Sample	Expanded	Sample	Expanded
**COMMUNITIES OF ORIGIN**							
**General hospitals**							
Patients with US residence in last year**		1222	1516	--	--	--	--
Repatriated due to illness	Patient survey	733	1061	--	--	--	--
	Staff survey	910	1129	119	492	1029	1621

Referral hospitals*							
Patients with US residence in last year**		596	736	--	--	--	--
Repatriated due to illness	Patient survey	298	368	--	--	298	368
	Staff survey	320	480	--	--	320	480

**US-MEXICO BORDER CITIES**							
Patients with US residence in last year**		887	1112	--	--	--	--
Repatriated due to illness	Patient survey	443	556	--	--	--	--
	Staff survey	746	908	215	2416	961	3324

**TOTAL**							
Patients with US residence in last year**		**2705**	**3364**	--	--	**--**	**--**
Repatriated due to illness	Patient survey	**1474**	**1985**	--	--	**--**	**--**
	Staff survey	**1976**	**2517**	**334**	**2908**	**2310**	**5425**

*No private referral hospitals data was considered in the study

**No information available for private hospitals

### Hospital admissions in the interior region

According to staff testimony, general hospitals in communities of origin cared for between 1 and 200 repatriated patients in 2008, for a total of 910 admissions (Table 2). The 7 repatriated patients observed through the patient survey are projected to 733 patients per year using the hospitals' average figure for patient bed-days of 2.98. These two figures are convergent and suggest a high degree of reliability. Expanding these figures to the universe of 33 public hospitals in the region, between 1,061 and 1,129 repatriated migrants per year demanded care, accounting for between 0.79% and 0.90% of total admissions. For every 10 repatriated sick migrants demanding hospital care in the interior region, 2.7 additional migrants demanded care, but returned to their communities of origin for reasons other than their health.

Staff of 16 private hospitals in communities of origin reported between 10 and 50 repatriated sick migrants for 2008 while in 4 hospitals no cases were reported. The median figure is 20 repatriations for a total of 215 in the sample. Projecting these figures to the 40 private hospitals in the region a total of 492 admissions occurred in 2008. Adding together the expanded public and private general hospital admissions for repatriated migrants in communities of origin, between 1,029 and 1,621 admissions occurred in 2008.

The referral hospitals observed in the interior region received a total of 320 repatriated patients in 2008, according to staff testimony. Only one repatriated patient was interviewed. These figures are projected to between 368 (based on patient observation) and 480 (based on staff interviews) admissions per year for all the referral hospitals in the region. These totals account for between 0.12% and 0.16% of referral hospital bed occupancy for 2008. Adding the total of public and private general hospital admissions to the referral hospital total, between 1429 and 2101 repatriated migrants demanded both levels of care in this region. Of them, 69.6% were attended in public hospitals and 30.3% in private hospitals.

### Hospital admissions in border cities

Staff in each of the 6 public hospitals visited in the US-Mexico border reported between 50 and 220 admissions from repatriated migrants and migrants injured or sick while crossing the border in 2008. Testimony in two of these hospitals was supported in administrative records kept to identify admitted indigents whose fees are exempted. Importantly, all repatriated migrants in these two hospitals were registered as indigents. A total of 746 repatriated sick migrants were reported in the sampled border public hospitals, which can be projected to 908 for the 10 Ministry of Health hospitals in the border region (Table 2). Patient surveys yielded 3 repatriated sick migrants across the 6 hospitals, projected to 556 admissions per year. The two sources are somewhat divergent, pointing to a range of between 556 and 908 admissions per year. These figures account for between 0.72% and 1.18% of public hospitals bed occupancy in border cities for 2008.

In the 6 private hospitals visited, between 30 and 60 repatriated sick migrants were admitted according to the staff survey in 2008 in each hospital, for a total of 215. Staff reported that most cases were of more wealthy migrants enjoying legal residence in the US. This figure was projected to 2,416 admissions in all the private hospitals in the border region.

Adding public and private hospital data for the US-Mexico border area, it can be estimated (using only the staff estimate for the case of private hospitals) that between 2972 and 3324 repatriated migrants were admitted in 2008, 27.3% from public and 72.7% from private hospitals.

### Admission diagnoses

A total of 12 repatriated sick migrants diagnoses were mentioned by staff as the most common across hospitals (table 3). The most frequently mentioned diagnosis is traumatisms, with 56.4% of hospitals mentioning it. It is followed by complications of diabetes (except chronic renal insufficiency), with 38.2% of hospitals mentioning it. Elective surgery followed with 36.4%. Referral hospitals in the interior region emphasized renal insufficiency, with three out of the four mentioning it, followed by cancer with two mentions. Public general hospitals in this region mention most frequently traumatism (80%) followed by diabetes (56%). Private hospitals in the interior region emphasize elective surgery diagnoses (57.1%) followed by diabetes (35.7%). Public hospitals along the border mention in all cases traumatisms, followed by animal bites and dehydration and respiratory diseases. All private hospitals along the border mention elective surgery, followed by diabetes, other chronic diseases and respiratory disease, all with 16.7%. HIV-AIDS is mentioned as a common diagnosis by a third of public general hospitals.

**Table 3:** Percentage of Hospitals Reporting a "Principal Diagnosis of Repatriated Migrant Patients", According to Type of Hospital and Region

DIAGNOSES MENTIONED AMONG PRINCIPAL		PUBLIC HOSPITALS				PRIVATE HOSPITALS		
		
				General				
							
	All Hospi-tals	All	Referral	Interior region	Border region	All	Interior region	Border region
Traumatism	**56.4**	83.9	100	80	100	5	7.1	0
Diabetes complications	**38.2**	48.4	25	56	0	30	35.7	16.7
Elective surgery	**36.4**	22.6	25	24	0	70	57.1	100
HIV-AIDS	**20**	32.3	0	32	33.3	5	7.1	0
Chronic renal failure	**18.2**	32.3	75	28	0	0	0	0
Cancer	**18.2**	29	50	28	0	5	7.1	0
Other chronic diseases	**14.5**	16.1	25	4	50	15	14.3	16.7
Respiratory diseases	**12.7**	19.4	0	8	66.7	5	0	16.7
Animal bites and dehydration	**7.3**	12.9	0	0	66.7	0	0	0
Psychiatric disorders	**3.6**	6.5	25	0	16.7	0	0	0

### Share of migrant hospitalization needs being met in Mexico

This measure was estimated with lower and upper bounds. For the lower bound, the migrant population likely to return to interior region hospitals if sick was estimated to be the number of migrants that had recently traveled from households in the interior region. The migrant household dependency factor of 0.767 was applied to the total 1,279,220 interior region households [17], giving a population of 981,162 migrants. For the upper bound the same procedure was used, except that only the 197,256 households that reported both travel and remittances were considered, for a migrant population of 151,671.

Given the hospitalization rate of Hispanics with low English proficiency in the US of 6.8% [8], a total of between 86,987 and 10,313 repatriated sick migrants are hospitalized yearly for the migrant population in each projection. The 1609 hospitalizations from repatriated sick migrants in public hospitals (observed through the staff survey) account for between 1.8% and 15.6% of total hospitalization needs. The observed 492 hospitalizations in private hospitals in the same region account for between 0.57% and 4.8% of the total hospitalization needs. Together, hospitals in the interior region take care of between 2.4% and 20.4% of expected hospitalizations. The remainder occur either in the US or Canada or in hospitals in Mexico outside the interior region.

## Discussion

The study of hospitalizations by repatriated migrants presented singular methodological difficulties. The lack of migration history in hospital records required the undertaking of a patient survey with low numbers of observations, although triangulation with a qualitative staff survey provided a sufficient degree of concordance. Given these limitations, the study provides a range of values for hospitalizations by migrants, attaining greater rigor for the interior region. Given the lack of sufficient data observed in the patient survey, the study was not able to analyze the determinants of demand for medical care.

The low percentage of total bed occupancy by migrants -between 0.79% and 0.90% for the interior region and between 0.72% and 1.18% at the border, suggest that there is still ample capacity to expand the supply of hospital services for this population group, even considering the possibility of high levels of current occupancy. Utilization of private hospitals by 30.3% of the total migrant demand in the interior region is somewhat higher than the national figure of 20.9% observed for the non-insured [18] and is congruent with the higher consumption of private care by migrant households noted by others [13].

The estimator for hospitalizations derived from the MEPS study of 6.8% for population over 18 is robust; indeed, the 2008 National Health Interview Survey shows that among the same age group answering in Spanish, 6.1% reported a hospital stay [19]. However, these figures include care for those over 65 and for women seeking maternity care. These populations have somewhat greater access to care in the US due to Medicaid eligibility or access to emergency services and are, therefore, less likely to return to Mexico to satisfy their needs. If maternity and elderly hospital needs were excluded from the study, the proportion of sick migrants repatriating to Mexico seeking hospital care would be higher. On the other hand, the utilization would tend to be lower if the healthy migrant effect is taken into consideration [20], whereby recent migrants have better health status than those with a greater degree of assimilation to the US and which are included in the MEPS hospitalization rate.

The estimated figures of between 2.4% and 20.4% of migrant hospitalization needs being satisfied in Mexico are likely to limit the bounds of this phenomenon. More research is required to assess the demographic and social factors that play a role in attracting patients to hospital care in Mexico. In any case, the medical care system does not need to fear being overwhelmed even if the return rate increased through insurance coverage or other reasons.

Seguro Popular is close to attaining universal coverage of households without employer-contributed health insurance. However, repatriated migrants will enjoy this benefit now only if they are the head of household or an economic dependent, or if they affiliate at the point of service. No data is available to ascertain the proportion of migrants that can claim Seguro Popular based on current rules and affiliation procedures. Furthermore, Seguro Popular covers a limited set of health conditions and excludes dialysis, a condition that is likely to be on high demand for repatriated migrants. Furthermore, Seguro Popular charges an annual fee that starts at USD 70 per household beyond the poverty line. The question arises as to the interest and capacity of Mexican migrants to affiliate to Seguro Popular while in the US, and the risk of being left out of health insurance in both countries. As migrants return, possibly in great numbers, the cherished target of attaining universal coverage for financial protection for health services may be elusive.

## Conclusions

Hospitals located in Mexico's high and very high migration intensity municipalities and in cities along the US-Mexico border are being used by repatriated sick migrants unable to satisfy their hospitalization needs abroad. Highest demand is for Ministry of Health hospitals in the interior region and for private hospitals in the border. In either case, migrants use a very low share of available capacity. However, up to 20% of migrant hospitalization needs are being satisfied by hospitals in the interior region. More research is required to ascertain the factors that attract migrants to hospitals in Mexico and to develop the managerial capacity to address their health needs with optimum quality.

The Mexican Constitution enshrines the Right to Health for all citizens in Mexico. This right should ensure access to health services in Mexico by migrants beyond the border that return given the difficulty in gaining access to services in the US [10]. The current insurance scheme for population in the informal sector of the economy in Mexico known as "Seguro Popular" should be reinforced through opportunities for affiliation beyond the border, as well as through identification of reliable and welcoming providers in Mexico. Portability and coverage of interventions should be redesigned to cater for the most important migrant health needs. Clinical and administrative records in hospitals should include migratory history. If diagnoses are related to a work injury or an occupational disease, hospitals in Mexico and migrants could claim reimbursement from state governments in the US through the Workers Compensation legislation.

Efforts should be made to improve bi-national medical networks as to ensure timely, quality referral and counter-referral procedures between hospitals and primary care. Mexican hospitals should actively welcome migrants and strive to provide services with the quality expected by them and by their households. Proposals for bi-national health insurance should focus attention on the important inflow of migrants into public hospitals in Mexico and on the need to offer health insurance policies that include options for migrant household members.

Research is urgently needed to ascertain the costs of care for migrants, the managerial processes that need to be put in place, the ethical and Constitutional obligations of the State to insure access to care in Mexico as well as the role that households in Mexico play to attract sick migrants and to facilitate access to medical care and to financial protection.

## Conflict of interests

The authors declare that they have no competing interests.

## Authors' contributions

Both authors participated in the planning and conception of the research questions and the study design. MAGB was the principal investigator of the study and primarily conceptualized the research. LASV was responsible for retrieving the data, and for analyzing the data. MAGB drafted the article, and all authors participated in interpreting the data and critically revising the manuscript for important intellectual content. All authors read and approved the revised manuscript.

## Pre-publication history

The pre-publication history for this paper can be accessed here:

http://www.biomedcentral.com/1471-2458/11/241/prepub

## References

[B1] Pew Research Center Mexican Immigrants in the United States, 2008. Fact sheet 2009 Washington, D.C.: Pew Hispanic Center

[B2] Colegio de la frontera norte, Consejo nacional de población, Secretaría de gobernación, Instituto nacional de migración Encuesta sobre migración en la frontera norte de México, 2005 Annual series 2007 México: CONAPO

[B3] Consejo Nacional de Población Migración residente en Estados Unidos, 2007 2008 México: CONAPO

[B4] Wallace S Access to health insurance & care among mexican bi-nationals: reality & potentialproceedings of Academy Health's 2007 Annual Research Meeting 2007 Orlando, Florida

[B5] Hansen E Donohoe M Health issues of migrant and seasonal farmworkers Journal of Health care for the Poor and Underserved 2003 14 2 153 164 12739296 10.1353/hpu.2010.0790

[B6] Ortega AN Fang H Perez VH Rizzo JA Carter-Pokras O Wallace SP Gelberg L Health care access, use of services, and experiences among undocumented mexican and other latinos Arch Intern Mec 2007 167 2354 2360 10.1001/archinte.167.21.2354 18039995

[B7] Secretaría de Salud Boletín de Información Estadística No. 28 2008 Sistema Nacional de Información en Salud. México

[B8] Brach C Chevarley F Demographics and Health Care Access and Utilization of Limited-English-Proficient and English-Proficient Hispanics 2008 The medical expenditure panel survey (MEPS) Washington, D.C.: Agency for healthcare research and quality, U.S. Department of health & human services 2008 Research finding #28

[B9] Landeck M Garza C Utilization of psysician health care services in Mexico by U.S. Hipanic border residents Health Marketing Quarterly 2002 20 3 16 10.1300/J026v20n01_02 12749595

[B10] Wallace S Mendez-Luck C Castañeda X Heading south: Why mexican immigrants in California seek health services in Mexico Med Care 2009 47 662 669 10.1097/MLR.0b013e318190cc95 19434002 PMC2711545

[B11] Vargas-Bustamante A Ojeda G Castaneda X Willingness to pay for cross-border health insurance between the United States and Mexico Health Affairs 2008 27 169 178 10.1377/hlthaff.27.1.169 18180492

[B12] Dirección General de Información en Salud Boletín de información estadística 2003 México: Secretaría de Salud

[B13] González-Block MA Robinson S de la Sierra LA González LM Olivares JC York P Salud Migrante: a proposal for binational Health insurance 2008 Cuernavaca: National Institute of Public Health

[B14] Tuirán R Fuentes C Ávila JL Índice de intensidad migratoria México-Estados Unidos 2000 2002 México: Consejo Nacional de Población

[B15] PC editores Informe sobre el universo de hospitales en la República Mexicana 2004 México

[B16] Princeton University and Universidad de Guadalajara Mexican Migrant Project 128 (file house) http://mmp.opr.princeton.edu [accessed 2010 Oct]

[B17] Instituto Nacional de Estadística y Geografía Conteo de población y vivienda 2005 2005 México: INEGI

[B18] Olaiz-Fernández G Rivera-Dommarco J Shamah-Levy T Rojas R Villalpando-Hernández S Hernández-Avila M Sepúlveda-Amor J Encuesta Nacional de Salud y Nutrición 2006 2006 Cuernavaca, México: Instituto Nacional de Salud Pública

[B19] Adams PF Heyman KM Vickerie JL Summary health statistics for the U.S. population. National Health Interview Survey, 2008 2009 10 243 Washington, D.C.: National Center for Health Statistics: Vital Health Stat 20821904

[B20] Pitkin Derose K Bahney BW Lurie N Escarce JJ Review: immigrants and health care access, quality, and cost Med Care Res Rev 2009 66 4 355 408 10.1177/1077558708330425 19179539

